# Supplementary material for: Genome-Wide Identification of Cyclophilin Gene Family in Cotton and Expression Analysis of the Fibre Development in Gossypium barbadense
Source: Int J Mol Sci. 2019 Jan 16;20(2):349. doi: 10.3390/ijms20020349 (PMC6359516; doi:10.3390/ijms20020349)
Supplement: Supplementary file 1 [file ijms-20-00349-s001.zip › ijms-423111-supplementary/Additional File 11ú║Table S7 The Sequences of qRT-PCR primers in this study..pdf]

Additional File 11：Table S7 The Sequences of qRT-PCR primers in this study.

Primer name	Primer Sequence	Primer name	Primer Sequence
Gb14-2-F	CACTGGCGAGAAAGGGGTA	Gb26-3-F	TGTCATAGAGGGCACTTCA
Gb14-2-R	AGCGAACTTGGCTCCATAGA	Gb26-3-R	ATGCTCCATTATCACGCTT
Gb16-2-F	TCTAAGGGAGAAAGGAGCCG	Gb26-4-F	TCACCCCAGACCAATAATCA
Gb16-2-R	TGGTGTTTGGTCCAGCATTG	Gb26-4-R	ACCTTTATACCCGAAACCCT
Gb18-2-F	CCGTAGCGGCAAACCTCTC	Gb27-1-F	TCAAATGCTCATAGCCCAA
Gb18-2-R	CCACTCCGTCTTGGTCGTG	Gb27-1-R	ATTCTGCCAACAGCCTTAC
Gb18-3-F	GTGAGAAAGGCGTCGGTCGTT	Gb27-2-F	CGACACCAACGGCTCACAAT
Gb18-3-R	CGGGTCTGGTGTGCTTCTTGA	Gb27-2-R	CTCTGCCTTCCTTCCGCTTC
Gb18-4-F	CTTCTTTGACATGGCGATC	Gb28-F	TTGATAGGGGGTTTGTAGCC
Gb18-4-R	GGACCAGTGTGCTTCTTGA	Gb28-R	CTCACGACGGGTAGGAAGTT
Gb18-5-F	TCTGTAACGGCGAGAAAG	Gb36-F	ATGAAAGTTTGGGAGTTGAC
Gb18-5-R	CGGTGTGCTTCTTGATGA	Gb36-R	TGAAGAAGTGGTTGGTGTTA
Gb18-6-F	GACTATCGGTGGTCAGCCC	Gb37-4-F	CCCGTTTTGCCTCTCTCATCC
Gb18-6-R	TTCTCGTCAGCGAACTTGG	Gb37-4-R	ACTCGTCCTTCCTCTCCCCCA
Gb18-8-F	ACTGGACCTGGTTGTCTATCA	Gb37-5-F	CAACAAGATGCCAACTGAGG
Gb18-8-R	ACTTTCGGAGCCTACTTTTTC	Gb37-5-R	GAAGAACTGAACCGAACACG
Gb19-1-F	GCAGATGTTGTTCCTAAGAC	Gb39-3-F	CCTTGCTTGTGCGATACTTG
Gb19-1-R	CACTGTTTGCCATAGAGAGA	Gb39-3-R	ACTAACCGTTTGACCTGCCT
Gb19-3-F	AGAAAGGCAAGGGTGCTAAG	Gb39-4-F	TCTCAAATGGTTCGTAGGC
Gb19-3-R	TGCTCTCCATCCAACCAACT	Gb39-4-R	ACCGATTCCAAAGACTCAA
Gb20-1-F	AATCCAAAAAATCCAATCG	Gb47-1-F	GCAGTTGGAGGGAGAGATTG
Gb20-1-R	TACATCCACCACCATCACC	Gb47-1-R	AGGGGTAACGACAGAGAAAG
Gb20-2-F	AATCCAAAAAACCCAATCGTA	Gb48-F	TGACCAACCAAGGGTAGAAA
Gb20-2-R	ACCATCACCCTTGAGGAAATC	Gb48-R	GAGCAAACTCCAACCGAACT
Gb21-4-F	AAGGGTGACACTGACAACTC	Gb58-F	TAGTGACCGCTCTGTAAAGG
Gb21-4-R	TGTGATGAAAAGCTCAAATG	Gb58-R	AACATTGGCACATACTCGCT
Gb23-F	CAGGGTTTTGATGATGATT	Gb62-F	AAGGCTGCTGCTACTAACC
Gb23-R	GTTTTAGGAACGGTTTTGC	Gb62-R	TTCTTGAGGCAATACACCC
Gb24-1-F	GTTATTGGATTGTATGGCGAA	Gb66-1-F	CCTGGTCTTTTATCTATGGC
Gb24-1-R	TATTATGCGATGGAATGGGGT	Gb66-1-R	CTTCAATGACTTCACCACAA
Gb24-2-F	TCCCAACCTAACTCATCTGCT	Gb70-F	TGAGGGTGGTGATTTTGTAA
Gb24-2-R	ATTCTCTCCACCGATTTCAAC	Gb70-R	AAGTATCGTTGCCCGTTCTA
Gb26-1-F	CTCACCCCAGACCAATAATCA	Gb77-F	AAAATAAGTTGAGAACGGGC
Gb26-1-R	AAAAGCCGAACCAGAGCACAG	Gb77-R	TCATCATTGTCACTGCTGCT
Gb26-2-F	TACAACAGAGCAAAAGGAAG	GbUBQ7-F	CCAGAAGGAATCCACTTTGC
Gb26-2-R	GTAGGTGGCATACACAAAAA	GbUBQ7-R	CCAGCTCACATCAGCATACG
