# Supplementary material for: Genome-Wide Identification of Cyclophilin Gene Family in Cotton and Expression Analysis of the Fibre Development in Gossypium barbadense
Source: Int J Mol Sci. 2019 Jan 16;20(2):349. doi: 10.3390/ijms20020349 (PMC6359516; doi:10.3390/ijms20020349)
Supplement: Supplementary file 1 [file ijms-20-00349-s001.zip › ijms-423111-supplementary/Additional File 1ú║Table S1 The CYPs gene family information in cottn.pdf]

Additional File 1: Table S1 The CYPs gene family information in cottn

Gene Name	Gene ID	Gene		Predicted polypeptide				
		Arabidopsis homology	Similarity (%)	Mw (kDa)	Length (aa)	Domain (CLD)	pI	Subcellular Location
GbCYP8	GOBAR_AA07343	AtCYP22-1	141/181	8.50	78	3-70	8.8	Cytoplasm(2.921)
GbCYP14-1	GOBAR_AA37002	AtCYP40	59/99	14.61	132	7-127	9.2	Cytoplasm(2.945)
GbCYP14-2	GOBAR_AA02739	AtCYP19-2	110/138	14.71	138	8-138	8.7	Cytoplasm(2.151)
GbCYP15	GOBAR_AA30180	AtCYP19-1	104/152	15.96	152	8-150	9.1	Cytoplasm(3.450)
GbCYP16-1	GOBAR_DD19905	AtCYP19-3	107/138	16.53	152	7-138	9.1	Cytoplasm(2.734)
GbCYP16-2	GOBAR_DD26079	AtCYP19-4	97/131	16.67	154	12-151	8.8	Cytoplasm(2.401)
GbCYP18-1	GOBAR_DD25583	AtCYP19-1	132/169	18.12	171	7-170	8.2	Cytoplasm(3.417)
GbCYP18-2	GOBAR_DD12369	AtCYP19-2	136/172	18.12	172	8-171	8.6	Cytoplasm(3.210)
GbCYP18-3	GOBAR_AA37830	AtCYP19-1	133/169	18.18	171	7-170	7.6	Cytoplasm(3.627)
GbCYP18-4	GOBAR_DD08058	AtCYP19-1	135/173	18.20	173	8-171	8.6	Cytoplasm(2.890)
GbCYP18-5	GOBAR_DD26548	AtCYP19-1	136/173	18.23	173	8-171	8.6	Cytoplasm(2.895)
GbCYP18-6	GOBAR_AA04404	AtCYP19-1	132/172	18.29	173	8-171	8.2	Cytoplasm(3.323)
GbCYP18-7	GOBAR_AA30181	AtCYP19-1	132/172	18.38	173	8-171	8.6	Cytoplasm(3.215)
GbCYP18-8	GOBAR_DD26547	AtCYP19-1	124/171	18.50	173	8-171	9.2	Cytoplasm(3.881)
GbCYP18-9	GOBAR_AA08474	AtCYP19-3	130/172	18.80	174	7-170	7.6	Cytoplasm(3.913)
GbCYP19-1	GOBAR_AA21449	AtCYP63	112/146	19.40	177	10-156	8.4	Cytoplasm(1.483)
GbCYP19-2	GOBAR_AA08313	AtCYP19-1	32/55	19.77	181	27-180	8.6	Cytoplasm(2.207)
GbCYP19-3	GOBAR_DD25587	AtCYP21-1	104/137	19.85	180	50-150	7.1	Cytoplasm(1.357) /Extracellular(1.327)
GbCYP20-1	GOBAR_DD01305	AtCYP22-1	157/184	20.49	189	25-188	8.4	Cytoplasm(2.421)
GbCYP20-2	GOBAR_AA24356	AtCYP22-1	158/184	20.57	189	25-188	8.4	Cytoplasm(2.114)
GbCYP20-3	GOBAR_AA31544	AtCYP22-1	158/180	20.68	191	27-190	8.3	Cytoplasm(2.267) /Nucleus(4.234)
GbCYP21-1	GOBAR_AA17890	AtCYP95	42/81	21.21	191	2-83	8.9	Nucleus(2.516)
GbCYP21-2	GOBAR_DD03339	AtCYP21-4	106/171	21.39	191	38-187	6.3	Cytoplasm(2.595)
GbCYP21-3	GOBAR_AA37755	AtCYP21-4	112/182	21.58	192	38-188	7.0	Cytoplasm(2.347)
GbCYP21-4	GOBAR_AA27853	AtCYP21-4	90/166	21.61	187	82-175	9.1	Mitochondria(1.532) /Extracellular(1.159)
GbCYP22	GOBAR_DD11460	AtCYP95	72/115	22.91	221	101-156	4.7	Cytoplasm(2.072)
GbCYP23	GOBAR_AA31887	AtCYP19-4	145/214	23.84	221	55-218	8.9	Cytoplasm(2.371)
GbCYP24-1	GOBAR_AA23113	AtCYP21-1	140/179	24.52	224	50-214	6.7	Cytoplasm(2.246)
GbCYP24-2	GOBAR_AA26140	AtCYP20-3	131/265	24.63	227	80-222	7.0	Chloroplast(1.069)
GbCYP26-1	GOBAR_AA14287	AtCYP20-2	152/261	26.01	240	82-234	9.6	Chloroplast(3.025)
GbCYP26-2	GOBAR_DD13607	AtCYP21-4	136/233	26.70	234	80-230	7.8	Mitochondria(1.603) /Nucleus(1.245)
GbCYP26-3	GOBAR_DD37745	AtCYP21-4	142/233	26.76	236	82-232	9.1	Mitochondria(1.776) /Nucleus(1.569)
GbCYP26-4	GOBAR_DD00684	AtCYP20-2	160/262	26.92	248	82-242	9.5	Chloroplast(2.870)
GbCYP27-1	GOBAR_DD34810	AtCYP20-3	149/264	27.08	250	93-245	8.4	Chloroplast(1.510) /Cytoplasm(1.100)
GbCYP27-2	GOBAR_AA19866	AtCYP19-4	151/205	27.81	255	64-252	9.1	Cytoplasm(1.910)
GbCYP28	GOBAR_AA29091	AtCYP23-1	170/214	28.07	249	29-186	5.6	Chloroplast(1.714) /Extracellular(1.026)
GbCYP29	GOBAR_AA33211	AtCYP28	152/245	29.71	268	80-224	6.4	Extracellular(2.187)
GbCYP34	GOBAR_DD01435	AtCYP26-2	155/246	34.89	320	100-291	8.1	Chloroplast(2.925)
GbCYP36	GOBAR_DD28495	AtCYP23-1	154/189	36.69	322	58-215	8.9	Cytoplasm(1.037) /Mitochondria(1.018)
GbCYP37-1	GOBAR_AA17121	AtCYP37	201/333	37.28	331	162-318	5.4	Extracellular(2.530)
GbCYP37-2	GOBAR_AA11120	AtCYP40	203/307	37.75	335	6-142	5.8	Cytoplasm(3.319)
GbCYP37-3	GOBAR_AA14886	AtCYP26-2	156/271	37.78	345	100-288	8.4	Chloroplast(3.175)
GbCYP37-4	GOBAR_DD24495	AtCYP37	216/333	37.84	343	164-320	5.4	Extracellular(1.865)

Additional File 1: Table S1 The CYP gene family information in cottn(continued)

Gene Name	Gene ID	Gene		Predicted polypeptide				
		Arabidopsis homology	Similarity (%)	Mw (kDa)	Length (aa)	Domain (CLD)	pI	Subcellular Location
GbCYP37-5	GOBAR_AA19718	AtCYP37	227/333	37.93	343	164-320	6.4	Extracellular(1.865)
GbCYP38	GOBAR_DD21364	AtCYP37	198/348	38.52	343	183-330	5.4	Extracellular(2.198)
GbCYP39-1	GOBAR_DD36199	AtCYP38	263/359	39.28	358	178-348	4.7	Cytoplasm(2.131)
GbCYP39-2	GOBAR_DD34612	AtCYP65	219/415	39.33	370	162-309	9.4	Mitochondria(2.238)
GbCYP39-3	GOBAR_AA36652	AtCYP37	212/348	39.58	358	179-335	5.2	Extracellular(1.502) /PlasmaMembrane (1.019)
GbCYP39-4	GOBAR_DD27607	AtCYP40	210/333	39.87	361	182-338	5.1	Extracellular(1.841)
GbCYP40-1	GOBAR_DD26347	AtCYP22-1	155/180	40.35	365	27-190	8.4	Cytoplasm(2.458)
GbCYP40-2	GOBAR_DD36127	AtCYP37	94/343	40.47	368	176-362	4.5	Cytoplasm(2.399)
GbCYP40-3	GOBAR_DD26754	AtCYP40	241/360	40.63	361	7-172	5.5	Cytoplasm(3.865)
GbCYP41-1	GOBAR_AA27859	AtCYP65	195/414	41.36	389	140-317	8.8	Nucleus(1.948)
GbCYP41-2	GOBAR_AA25604	AtCYP40	248/360	41.39	371	8-173	5.7	Cytoplasm(2.967)
GbCYP42-1	GOBAR_AA16016	AtCYP38	321/385	42.57	388	196-382	4.6	Cytoplasm(2.130)
GbCYP42-2	GOBAR_AA40635	AtCYP37	169/221	42.64	388	216-381	8.5	Chloroplast(3.209)
GbCYP43-1	GOBAR_DD26812	AtCYP40	222/386	43.15	385	7-173	5.4	Cytoplasm(4.082)
GbCYP43-2	GOBAR_AA09401	AtCYP40	223/386	43.35	387	7-173	5.6	Cytoplasm(3.822)
GbCYP43-3	GOBAR_DD04774	AtCYP57	134/191	43.55	388	14-168	8.6	Nucleus(3.590)
GbCYP47-1	GOBAR_AA24967	AtCYP59	262/325	47.30	427	77-235	4.8	Cytoplasm(2.377)
GbCYP47-2	GOBAR_AA36023	AtCYP40	248/376	47.40	419	8-173	8.2	Cytoplasm(3.140)
GbCYP48	GOBAR_AA12952	AtCYP57	139/210	48.92	435	14-168	9.0	Nucleus(3.309)
GbCYP49-1	GOBAR_DD27655	AtCYP40	246/360	49.02	438	10-175	8.4	Cytoplasm(2.109) /Nucleus(1.687)
GbCYP49-2	GOBAR_AA22689	AtCYP37	298/424	49.87	458	286-451	6.1	Chloroplast(3.305)
GbCYP58	GOBAR_AA15867	AtCYP71	426/520	58.51	519	370-516	6.8	Cytoplasm(1.712) /Nucleus(1.043)
GbCYP61	GOBAR_AA36607	AtCYP38	297/394	61.02	549	245-436	5.8	Cytoplasm(2.471)
GbCYP62	GOBAR_DD27614	AtCYP63	148/183	62.55	570	10-174	10.6	Nucleus(3.596)
GbCYP63	GOBAR_DD35486	AtCYP95	98/174	63.51	564	50-137	11.4	Nucleus(3.892)
GbCYP66-1	GOBAR_AA24902	AtCYP95	136/180	66.32	583	22-180	11.1	Nucleus(4.234)
GbCYP66-2	GOBAR_DD21100	AtCYP40	240/360	66.94	596	7-169	7.2	Nucleus(3.554)
GbCYP70	GOBAR_AA24903	AtCYP95	91/174	70.89	629	10-125	11.2	Nucleus(4.065)
GbCYP72	GOBAR_AA31583	AtCYP63	144/175	72.04	654	3-167	10.7	Nucleus(3.865)
GbCYP77	GOBAR_DD35485	AtCYP95	135/180	77.47	696	22-180	11.3	Nucleus(3.838)
GbCYP79	GOBAR_AA36544	AtCYP59	324/504	79.02	686	2-161	6.9	Nucleus(4.693)
GbCYP142	GOBAR_AA09575	AtCYP21-4	114/212	142.5	1256	1102-1252	9.2	Nucleus(2.585)
GhCYP12	Gh_Sca140771G01	AtCYP19-2	86/112	12.06	112	1-112	6.9	Cytoplasm(2.286)
GhCYP17	Gh_A08G1194	AtCYP18-1	139/160	17.40	160	2-153	8.4	Cytoplasm(2.156)
GhCYP18-1	Gh_A01G1747	AtCYP18-2	75/160	18.02	164	11-162	7.8	Cytoplasm(1.453) /Chloroplast(1.152) /Mitochondria(1.013)
GhCYP18-2	Gh_D12G2822	AtCYP19-2	136/172	18.12	172	8-171	8.6	Cytoplasm(3.224)
GhCYP18-3	Gh_A01G0031	AtCYP19-1	130/169	18.18	171	7-170	7.7	Cytoplasm(3.443)
GhCYP18-4	Gh_D01G0030	AtCYP19-1	130/169	18.22	171	7-170	6.8	Cytoplasm(3.466)
GhCYP18-5	Gh_A01G1361	AtCYP19-1	136/173	18.23	173	8-171	8.6	Cytoplasm(2.908)
GhCYP18-6	Gh_D01G1605	AtCYP19-1	135/173	18.26	173	8-171	8.6	Cytoplasm(2.622)
GhCYP18-7	Gh_A04G1047	AtCYP19-1	133/172	18.28	173	8-171	8.3	Cytoplasm(3.279)
GhCYP18-8	Gh_A04G1046	AtCYP19-2	126/173	18.46	173	8-171	9.5	Cytoplasm(3.671)
GhCYP18-9	Gh_D04G1620	AtCYP19-1	124/171	18.50	173	8-171	9.3	Cytoplasm(3.887)
GhCYP18-10	Gh_Sca006066G02	AtCYP18-2	118/150	18.62	170	11-160	9.1	Cytoplasm(1.190) /Nucleus(1.005)
GhCYP18-11	Gh_D13G1093	AtCYP19-3	129/172	18.76	174	7-170	6.9	Cytoplasm(3.863)

Additional File 1: Table S1 The CYP gene family information in cottn(continued)

Gene Name	Gene ID	Gene		Predicted polypeptide				
		Arabidopsis homology	Similarity (%)	Mw (kDa)	Length (aa)	Domain (CLD)	pI	Subcellular Location
GhCYP18-12	Gh_A13G0846	AtCYP19-3	130/172	18.80	174	7-170	7.6	Cytoplasm(3.882)
GhCYP19-1	Gh_A12G1281	AtCYP21-4	29/48	19.26	177	72-165	5.8	Extracellular(1.276) Chloroplast(1.228)
GhCYP19-2	Gh_A10G1687	AtCYP22-1	142/181	19.72	181	27-180	8.7	Cytoplasm(2.296)
GhCYP20-1	Gh_A05G3461	AtCYP22-1	158/184	20.44	188	24-187	8.4	Cytoplasm(2.183)
GhCYP20-2	Gh_D04G1937	AtCYP22-1	158/184	20.52	189	25-188	8.4	Cytoplasm(2.357)
GhCYP20-3	Gh_D10G1953	AtCYP22-1	158/180	20.63	191	27-190	8.4	Cytoplasm(2.328)
GhCYP20-4	Gh_A10G1682	AtCYP22-1	159/180	20.66	191	27-190	8.4	Cytoplasm(2.303)
GhCYP23-1	Gh_D10G2442	AtCYP19-4	146/214	23.84	221	55-218	8.9	Cytoplasm(2.452)
GhCYP23-2	Gh_A10G2121	AtCYP19-4	145/214	23.87	221	55-218	8.9	Cytoplasm(2.377)
GhCYP24-1	Gh_A01G0027	AtCYP21-1	140/179	24.52	224	50-214	6.7	Cytoplasm(2.244)
GhCYP24-2	Gh_A03G0865	AtCYP23-1	171/214	24.52	216	29-186	5.9	Chloroplast(1.586)
GhCYP24-3	Gh_D01G0026	AtCYP21-1	137/174	24.59	224	50-214	6.7	Cytoplasm(2.114)
GhCYP26-1	Gh_D02G0593	AtCYP21-4	141/235	26.27	234	80-230	9.3	Mitochondria(2.060)
GhCYP26-2	Gh_A09G0853	AtCYP20-1	145/186	26.32	246	41-205	9.5	Mitochondria(1.673) /Cytoplasm(1.349)
GhCYP26-3	Gh_A02G0528	AtCYP21-4	140/233	26.50	235	81-231	9.3	Mitochondria(1.692)
GhCYP26-4	Gh_D11G1133	AtCYP21-4	136/233	26.71	234	80-230	8.5	Mitochondria(1.638) /Nucleus(1.260)
GhCYP26-5	Gh_D08G0452	AtCYP21-4	141/233	26.74	236	82-232	8.9	Mitochondria(1.805) /Nucleus(1.426)
GhCYP26-6	Gh_A11G0987	AtCYP21-4	135/233	26.75	234	80-230	8.5	Mitochondria(1.729) /Nucleus(1.126)
GhCYP26-7	Gh_A08G0354	AtCYP21-4	142/233	26.91	236	82-232	8.7	Mitochondria(1.669)
GhCYP28-1	Gh_Sca004880G02	AtCYP20-2	168/262	28.08	259	93-253	9.8	Chloroplast(3.099)
GhCYP28-2	Gh_D01G0206	AtCYP20-2	167/262	28.10	259	93-253	9.7	Chloroplast(3.043)
GhCYP28-3	Gh_D02G1247	AtCYP23-1	173/219	28.39	249	58-215	7.7	Chloroplast(1.759)
GhCYP28-4	Gh_A12G2539	AtCYP40	161/253	28.55	254	1-65	5.8	Cytoplasm(3.044)
GhCYP29	Gh_D08G1359	AtCYP20-3	151/288	29.99	276	119-271	8.6	Chloroplast(1.426) /Extracellular(1.333)
GhCYP30-1	Gh_A05G0642	AtCYP63	94/131	30.03	273	10-110	8.6	Cytoplasm(2.575)
GhCYP30-2	Gh_D12G0852	AtCYP40	127/239	30.49	269	7-144	6.2	Cytoplasm(3.812)
GhCYP30-3	Gh_A08G1077	AtCYP20-3	151/288	30.98	284	119-279	8.4	Chloroplast(1.535) /Extracellular(1.140) /PlasmaMembrane(1.041)
GhCYP31-1	Gh_D06G0456	AtCYP28	169/246	31.85	289	81-245	6.8	Extracellular(2.173)
GhCYP31-2	Gh_A06G0418	AtCYP28	171/246	31.88	289	81-245	6.4	Extracellular(2.185)
GhCYP34-1	Gh_A03G0499	AtCYP26-2	156/247	34.95	321	100-291	8.7	Chloroplast(2.918)
GhCYP34-2	Gh_D03G1033	AtCYP26-2	155/247	34.98	321	100-291	8.2	Chloroplast(2.995)
GhCYP35	Gh_D11G0793	AtCYP37	189/310	35.98	322	153-309	5.4	Extracellular(1.445) /Cytoplasm(1.280) /Lysosomal(1.149)
GhCYP37-1	Gh_D09G1874	AtCYP37	216/333	37.84	343	164-320	5.5	Extracellular(2.002)
GhCYP37-2	Gh_A09G1765	AtCYP37	227/333	37.93	343	164-320	6.0	Extracellular(1.899)
GhCYP37-3	Gh_A08G1470	AtCYP37	212/333	37.95	343	164-320	5.4	Extracellular(1.770)
GhCYP37-4	Gh_D08G1766	AtCYP37	210/333	37.96	343	164-320	5.5	Extracellular(1.873)
GhCYP40-1	Gh_A10G0832	AtCYP40	235/361	40.05	360	7-173	6.0	Cytoplasm(3.783)
GhCYP40-2	Gh_D10G0925	AtCYP40	231/361	40.26	362	7-173	6.4	Cytoplasm(3.846)
GhCYP40-3	Gh_D03G0186	AtCYP40	239/360	40.62	361	7-172	5.5	Cytoplasm(3.862)
GhCYP41	Gh_A02G1526	AtCYP40	250/370	41.91	371	7-182	6.2	Cytoplasm(3.397)

Additional File 1: Table S1 The CYP gene family information in cottn(continued)

Gene Name	Gene ID	Gene		Predicted polypeptide				
		Arabidopsis homology	Similarity (%)	Mw (kDa)	Length (aa)	Domain (CLD)	pI	Subcellular Location
GhCYP44-1	Gh_A11G0678	AtCYP37	198/395	44.08	393	224-380	5.6	Extracellular(1.744) /Cytoplasm(1.203)
GhCYP44-2	Gh_A13G0333	AtCYP40	248/360	44.56	399	8-173	6.0	Cytoplasm(2.859)
GhCYP45-1	Gh_D13G0372	AtCYP40	245/360	45.43	403	28-193	6.7	Cytoplasm(2.631)
GhCYP45-2	Gh_A03G1688	AtCYP40	248/360	45.89	406	8-173	8.7	Cytoplasm(3.119)
GhCYP46	Gh_D02G2108	AtCYP40	253/360	46.43	410	8-173	8.6	Cytoplasm(2.895)
GhCYP47	Gh_A09G0254	AtCYP38	323/443	47.90	435	244-427	4.9	Cytoplasm(2.338)
GhCYP48	Gh_D09G0253	AtCYP38	312/404	48.05	436	245-425	4.9	Cytoplasm(2.504)
GhCYP49-1	Gh_D07G2233	AtCYP38	326/428	49.76	452	261-446	5.1	Chloroplast(3.246)
GhCYP49-2	Gh_Sca004717G03	AtCYP37	296/424	49.88	458	286-451	6.4	Chloroplast(3.297)
GhCYP49-3	Gh_Sca004717G12	AtCYP37	311/464	49.90	458	286-451	5.9	Chloroplast(3.497)
GhCYP49-4	Gh_A07G2012	AtCYP38	329/426	49.98	452	261-446	5.2	Chloroplast(2.914)
GhCYP55	Gh_D06G2331	AtCYP57	290/509	55.90	496	14-168	7.6	Nucleus(4.038)
GhCYP56	Gh_A06G0767	AtCYP57	290/510	56.10	497	14-168	8.4	Nucleus(3.722)
GhCYP65-1	Gh_A12G0709	AtCYP95	387/602	65.33	596	350-501	8.2	Mitochondria(1.387) /Nucleus(1.298)
GhCYP65-2	Gh_D12G0724	AtCYP65	398/604	65.39	596	350-501	8.2	Mitochondria(1.346) /Nucleus(1.346) /Cytoplasm(1.091)
GhCYP67	Gh_A08G1670	AtCYP63	144/175	67.99	619	3-167	10.8	Nucleus(4.008)
GhCYP70-1	Gh_A07G0986	AtCYP71	500/585	70.00	621	467-618	6.5	Cytoplasm(2.381)
GhCYP70-2	Gh_D07G1064	AtCYP71	499/585	70.04	621	467-618	6.6	Cytoplasm(2.425)
GhCYP70-3	Gh_A05G4019	AtCYP59	314/429	70.47	611	2-161	5.9	Nucleus(4.506)
GhCYP70-4	Gh_D05G0033	AtCYP59	313/429	70.56	611	2-161	5.7	Nucleus(4.445)
GhCYP72	Gh_D08G2018	AtCYP63	141/175	72.12	654	3-167	10.6	Nucleus(3.882)
GhCYP89-1	Gh_A07G0324	AtCYP95	132/174	89.29	790	10-174	11.5	Nucleus(3.700)
GhCYP89-2	Gh_D07G0381	AtCYP95	132/174	89.36	788	110-174	11.5	Nucleus(3.694)
GhCYP91	Gh_D07G0382	AtCYP95	130/182	91.13	806	22-182	11.5	Nucleus(3.676)
GhCYP92	Gh_A07G0325	AtCYP95	136/174	92.89	828	10-174	11.3	Nucleus(3.792)
GaCYP15	Cotton_A_15290	AtCYP19-2	115/150	15.65	149	1-148	6.8	Cytoplasm(3.175)
GaCYP17	Cotton_A_31267	AtCYP18-1	140/159	17.37	160	2-153	7.9	Cytoplasm(2.410)
GaCYP18-1	Cotton_A_10168	AtCYP18-2	129/164	18.02	164	11-162	7.8	Cytoplasm(1.453) /Chloroplast(1.152) /Mitochondria(1.013)
GaCYP18-2	Cotton_A_11089	AtCYP63	113/135	18.15	166	10-145	8.2	Cytoplasm(2.047)
GaCYP18-3	Cotton_A_00237	AtCYP19-1	133/169	18.18	171	7-170	7.6	Cytoplasm(3.487)
GaCYP18-4	Cotton_A_33740	AtCYP19-1	136/173	18.23	173	8-171	8.6	Cytoplasm(2.908)
GaCYP18-5	Cotton_A_08910	AtCYP19-2	135/172	18.26	173	8-171	8.2	Cytoplasm(3.454)
GaCYP18-6	Cotton_A_08911	AtCYP19-2	126/173	18.45	173	8-171	9.5	Cytoplasm(3.704)
GaCYP18-7	Cotton_A_39238	AtCYP19-3	130/172	18.80	174	7-170	7.6	Cytoplasm(3.882)
GaCYP20-1	Cotton_A_20244	AtCYP22-1	159/184	20.55	189	25-188	8.4	Cytoplasm (2.211)
GaCYP20-2	Cotton_A_15137	AtCYP22-1	158/18	20.68	191	27-190	8.3	Cytoplasm(2.271)
GaCYP21	Cotton_A_15133	AtCYP22-1	148/183	21.20	194	27-193	8.4	Cytoplasm(2.530)
GaCYP22	Cotton_A_21118	AtCYP20-1	152/183	22.19	206	41-203	9.4	Cytoplasm(1.992) /Mitochondria(1.529)
GaCYP23	Cotton_A_15858	AtCYP19-4	145/214	23.93	221	55-218	8.6	Cytoplasm(2.455)
GaCYP24-1	Cotton_A_00242	AtCYP21-1	140/179	24.46	224	50-214	6.4	Cytoplasm(2.167)
GaCYP24-2	Cotton_A_21779	AtCYP21-4	128/232	24.78	220	81-215	9.7	Mitochondria(2.427)
GaCYP24-3	Cotton_A_35717	AtCYP23-1	172/219	24.92	220	29-186	6.1	Chloroplast(1.669)
GaCYP26	Cotton_A_12588	AtCYP21-4	142/233	26.90	236	82-232	8.9	Mitochondria(1.646)
GaCYP27	Cotton_A_27185	AtCYP20-3	154/265	27.62	255	93-250	8.1	Chloroplast(1.436) /Cytoplasm(1.148)

Additional File 1: Table S1 The CYP gene family information in cottn(continued)

Gene Name	Gene ID	Gene			Predicted polypeptide			
		Arabidopsis Homology	Similarity (%)	Mw (kDa)	Length (aa)	Domain (CLD)	pI	Subcellular Location
GaCYP30	Cotton_A_23212	AtCYP21-4	135/267	30.60	268	80-211	7.7	PlasmaMembrane(1.589)
GaCYP31	Cotton_A_08379	AtCYP28	170/246	31.92	289	81-245	6.4	Extracellular(2.135)
GaCYP34	Cotton_A_28467	AtCYP26-2	156/246	34.92	320	100-291	8.2	Chloroplast(3.358)
GaCYP37-1	Cotton_A_07667	AtCYP37	201/333	37.30	331	162-318	5.5	Extracellular(2.404)
GaCYP37-2	Cotton_A_16591	AtCYP37	226/333	37.66	341	164-320	5.8	Extracellular(2.076)
GaCYP37-3	Cotton_A_13181	AtCYP37	212/333	37.90	343	164-320	5.4	Extracellular(1.997)
GaCYP40-1	Cotton_A_30789	AtCYP40	236/360	40.29	361	7-172	6.0	Cytoplasm(3.485)
GaCYP40-2	Cotton_A_35705	AtCYP40	232/361	40.29	362	7-173	6.6	Cytoplasm(3.786)
GaCYP40-3	Cotton_A_01679	AtCYP40	243/360	40.58	361	7-172	5.7	Cytoplasm(3.721)
GaCYP41	Cotton_A_19419	AtCYP40	248/360	41.39	371	8-173	5.7	Cytoplasm(2.931)
GaCYP45	Cotton_A_00780	AtCYP40	250/360	45.51	403	8-173	8.6	Cytoplasm(3.210)
GaCYP47	Cotton_A_10206	AtCYP38	322/443	47.88	435	244-427	4.9	Cytoplasm(2.290)
GaCYP49	Cotton_A_28873	AtCYP38	328/425	49.89	451	260-445	5.1	Chloroplast(2.914)
GaCYP50	Cotton_A_36132	AtCYP37	298/427	50.96	468	286-451	6.4	Chloroplast(3.374)
GaCYP56	Cotton_A_21009	AtCYP57	291/510	56.09	497	14-168	8.1	Nucleus(3.783)
GaCYP65	Cotton_A_34433	AtCYP65	388/602	65.40	596	350-501	8.3	Nucleus(1.464) /Mitochondria(1.301)
GaCYP70	Cotton_A_09572	AtCYP71	501/585	70.00	621	467-618	6.5	Chloroplast (2.449)
GaCYP71	Cotton_A_19569	AtCYP63	142/175	71.73	650	3-167	10.7	Nucleus(3.892)
GaCYP77	Cotton_A_00039	AtCYP20-2	151/193	77.20	704	538-698	6.7	PlasmaMembrane(1.104) /Nucleus(1.072) /Cytoplasm(1.036)
GaCYP89-1	Cotton_A_03540	AtCYP95	135/174	89.10	788	10-174	11.5	Nucleus(3.679)
GaCYP89-2	Cotton_A_03541	AtCYP95	132/174	89.80	795	10-174	11.5	Nucleus(3.702)
GrCYP18-1	Cotton_D_gene_10021857	AtCYP18-2	129/164	18.03	164	11-162	7.8	Cytoplasm(1.511) /Chloroplast(1.003)
GrCYP18-2	Cotton_D_gene_10038393	AtCYP19-2	136/172	18.12	172	8-171	8.6	Cytoplasm(3.224)
GrCYP18-3	Cotton_D_gene_10018166	AtCYP19-1	133/169	18.18	171	7-170	7.6	Cytoplasm(3.487)
GrCYP18-4	Cotton_D_gene_10038634	AtCYP19-1	134/173	18.24	173	8-171	8.6	Cytoplasm(2.879)
GrCYP18-5	Cotton_D_gene_10025556	AtCYP19-1	133/172	18.28	173	8-171	8.2	Cytoplasm(3.279)
GrCYP18-6	Cotton_D_gene_10025555	AtCYP19-1	124/171	18.48	173	8-171	9.1	Cytoplasm(4.063)
GrCYP18-7	Cotton_D_gene_10035185	AtCYP19-3	130/172	18.77	174	7-170	7.6	Cytoplasm(3.873)
GrCYP20	Cotton_D_gene_10016660	AtCYP22-1	158/184	20.52	189	25-188	8.4	Cytoplasm(2.357)
GrCYP22	Cotton_D_gene_10006562	AtCYP19-4	156/186	22.20	207	41-204	9.4	Cytoplasm(1.797) /Mitochondria(1.796)
GrCYP23-1	Cotton_D_gene_10013011	AtCYP20-1	56/103	23.41	210	145-202	9.1	PlasmaMembrane(3.901)
GrCYP23-2	Cotton_D_gene_10009280	AtCYP19-4	147/214	23.87	221	55-218	8.9	Cytoplasm(2.511)
GrCYP24-1	Cotton_D_gene_10005803	AtCYP21-4	122/210	24.55	219	80-214	9.7	Mitochondria(2.462)
GrCYP24-2	Cotton_D_gene_10018171	AtCYP21-1	142/179	24.59	224	50-214	6.7	Cytoplasm(2.205)

Additional File 1: Table S1 The CYP gene family information in cottn(continued)

Gene Name	Gene ID	Gene		Predicted polypeptide				
		Arabidopsis Homology	Similarity (%)	Mw (kDa)	Length (aa)	Domain (CLD)	pI	Subcellular Location
GrCYP25	Cotton_D_gene_100 37925	AtCYP23-1	175/220	25.14	222	29-186	7.0	Chloroplast(1.374) /Extracellular(1.031)
GrCYP26	Cotton_D_gene_100 35822	AtCYP21-4	136/233	26.66	233	80-229	8.4	Mitochondria(1.608) /Nucleus(1.181)
GrCYP27	Cotton_D_gene_100 04071	AtCYP20-3	149/264	27.10	250	93-245	8.4	Chloroplast(1.422) /Cytoplasm(1.142)
GrCYP28	Cotton_D_gene_100 17975	AtCYP20-2	167/262	28.10	259	93-253	9.6	Chloroplast(3.043)
GrCYP29	Cotton_D_gene_100 07881	AtCYP21-4	140/233	29.32	259	105-255	6.7	Nucleus(1.626) /Cytoplasm(1.022)
GrCYP31	Cotton_D_gene_100 04432	AtCYP28	170/246	31.85	289	81-245	6.3	Extracellular(1.966) /Nucleus(1.359)
GrCYP34	Cotton_D_gene_100 32360	AtCYP26-2	155/246	34.98	320	100-291	8.6	Chloroplast(3.120)
GrCYP37-1	Cotton_D_gene_100 15464	AtCYP37	217/333	37.80	343	164-320	5.4	Extracellular(1.912)
GrCYP37-2	Cotton_D_gene_100 37715	AtCYP37	212/333	37.98	343	164-320	5.5	Extracellular(2.090)
GrCYP40-1	Cotton_D_gene_100 40439	AtCYP40	238/360	40.36	361	7-172	6.5	Cytoplasm(3.451)
GrCYP40-2	Cotton_D_gene_100 20262	AtCYP22-1	155/180	40.43	365	27-190	8.7	Cytoplasm(2.292)
GrCYP40-3	Cotton_D_gene_100 04591	AtCYP40	240/360	40.57	361	7-172	5.5	Cytoplasm(3.848)
GrCYP41	Cotton_D_gene_100 32672	AtCYP37	201/373	41.55	371	202-358	5.3	Extracellular(1.705) /Cytoplasm(1.588)
GrCYP42-1	Cotton_D_gene_100 30327	AtCYP40	224/383	42.87	382	7-173	5.3	Cytoplasm(4.060)
GrCYP42-2	Cotton_D_gene_100 29383	AtCYP40	246/360	42.94	385	10-175	6.1	Cytoplasm(2.918)
GrCYP43	Cotton_D_gene_100 16497	AtCYP40	255/360	43.52	389	8-173	6.2	Cytoplasm(2.948)
GrCYP47	Cotton_D_gene_100 14459	AtCYP38	321/443	47.83	435	245-424	4.9	Cytoplasm(2.235) /Chloroplast(1.808)
GrCYP49-1	Cotton_D_gene_100 19448	AtCYP38	326/428	49.65	451	260-445	5.1	Chloroplast(3.391)
GrCYP49-2	Cotton_D_gene_100 12625	AtCYP37	311/464	49.92	458	286-451	6.1	Chloroplast(3.381)
GrCYP57	Cotton_D_gene_100 26358	AtCYP57	288/525	57.65	512	14-168	6.4	Nucleus(3.722)
GrCYP63	Cotton_D_gene_100 12967	AtCYP71	478/564	63.61	564	410-561	7.2	Cytoplasm(2.239)
GrCYP65	Cotton_D_gene_100 23324	AtCYP65	393/602	65.30	596	350-501	8.1	Mitochondria(1.319) /Nucleus(1.334) /Cytoplasm(1.126)
GrCYP72-1	Cotton_D_gene_100 15996	AtCYP59	315/429	72.49	627	2-161	5.7	Nucleus(4.369)
GrCYP72-2	Cotton_D_gene_100 05130	AtCYP63	146/183	72.83	659	10-174	10.8	Nucleus(3.901)
GrCYP90	Cotton_D_gene_100 09439	AtCYP95	135/174	90.62	801	10-174	11.5	Nucleus(3.673)
