# Supplementary material for: Genome-Wide Identification of Cyclophilin Gene Family in Cotton and Expression Analysis of the Fibre Development in Gossypium barbadense
Source: Int J Mol Sci. 2019 Jan 16;20(2):349. doi: 10.3390/ijms20020349 (PMC6359516; doi:10.3390/ijms20020349)
Supplement: Supplementary file 1 [file ijms-20-00349-s001.zip › ijms-423111-supplementary/Additional File 5ú║Table S2 Analysis and distribution of conserved motifs of CYP proteins in cotton (2).pdf]

Additional File 5：Table S2 Analysis and distribution of conserved motifs of CYP proteins in cotton.

Motif	Width	E value	Best possible match
1	24	8.7e-2849	MANAGPNTNGSQFFITTTKTEWLD
2	22	1.1e-2671	PLHYKGSRFHRVIKGFMIQGGD
3	27	1.2e-2668	AGRIVMELFADVVPKTAENFRALCTGE
4	32	6.1e-2843	FTAGBGTGGESIYGLKFADENFILKHTGPGVL
5	15	5.9e-1598	KHVVFGKVVKGMDVV
6	15	8.3e-981	GRPTKPVVIADCGEL
7	15	5.1e-921	KNPKVFFDISIGGZP
8	23	6.4e-712	GDITIELFPDNAPKTVDNFLDLC
9	32	1.1e-724	EQYKKQDYKIALRKYRKALRYLDICWELEGID
10	32	1.9e-738	GALLDTDFAIRDGEBNVKALFRQGQAHMALND
11	32	1.1e-716	DDGISNFFKDGDIYPDWPADLDEKPDEJSWWM
12	32	3.7e-879	PLEPNGGGIKKELAAAMKKIAERRDAEKRAYS
13	21	6.2e-547	GGRSIKGSIFTBESAKKLKLG
14	32	2.7e-577	LAPSKKDKGPEVIWTSGIVFGKLEGIVELRTE
15	29	2.5e-580	AESRGZDWDLKGKHIKQLPYSPPHALIQL
