# Supplementary material for: Genome-Wide Identification of Cyclophilin Gene Family in Cotton and Expression Analysis of the Fibre Development in Gossypium barbadense
Source: Int J Mol Sci. 2019 Jan 16;20(2):349. doi: 10.3390/ijms20020349 (PMC6359516; doi:10.3390/ijms20020349)
Supplement: Supplementary file 1 [file ijms-20-00349-s001.zip › ijms-423111-supplementary/Additional File 7ú║Table S3 Segmentally duplicated GbCYP gene pairs.pdf]

Additional File 7：Table S3 Segmentally duplicated GbCYP gene pairs

Gene Name	Gene ID	Gene Name	Gene ID
GbCYP14-1	GOBAR_AA37002	GbCYP40-3	GOBAR_DD26754
GbCYP14-2	GOBAR_AA02739	GbCYP18-2	GOBAR_DD12369
GbCYP15	GOBAR_AA30180	GbCYP18-8	GOBAR_DD26547
GbCYP18-3	GOBAR_AA37830	GbCYP18-1	GOBAR_DD25583
GbCYP18-4	GOBAR_DD08058	GbCYP18-2	GOBAR_DD12369
GbCYP18-6	GOBAR_AA04404	GbCYP18-4	GOBAR_DD08058
GbCYP18-7	GOBAR_AA30181	GbCYP18-5	GOBAR_DD26548
GbCYP18-8	GOBAR_DD26547	GbCYP18-2	GOBAR_DD12369
GbCYP18-9	GOBAR_AA08474	GbCYP16-1	GOBAR_DD19905
GbCYP19-2	GOBAR_AA08313	GbCYP20-1	GOBAR_DD01305
GbCYP19-2	GOBAR_AA08313	GbCYP40-1	GOBAR_DD26347
GbCYP20-1	GOBAR_DD01305	GbCYP40-1	GOBAR_DD26347
GbCYP20-2	GOBAR_AA24356	GbCYP19-2	GOBAR_AA08313
GbCYP20-2	GOBAR_AA24356	GbCYP20-1	GOBAR_DD01305
GbCYP21-3	GOBAR_AA37755	GbCYP21-2	GOBAR_DD03339
GbCYP21-4	GOBAR_AA27853	GbCYP26-3	GOBAR_DD37745
GbCYP23	GOBAR_AA31887	GbCYP16-2	GOBAR_DD26079
GbCYP24-1	GOBAR_AA23113	GbCYP19-3	GOBAR_DD25587
GbCYP26-1	GOBAR_AA14287	GbCYP26-4	GOBAR_DD00684
GbCYP37-1	GOBAR_AA17121	GbCYP38	GOBAR_DD21364
GbCYP37-2	GOBAR_AA11120	GbCYP40-3	GOBAR_DD26754
GbCYP37-3	GOBAR_AA14886	GbCYP34	GOBAR_DD01435
GbCYP37-5	GOBAR_AA19718	GbCYP37-4	GOBAR_DD24495
GbCYP39-3	GOBAR_AA36652	GbCYP37-5	GOBAR_AA19718
GbCYP39-3	GOBAR_AA36652	GbCYP37-4	GOBAR_DD24495
GbCYP40-2	GOBAR_DD36127	GbCYP39-1	GOBAR_DD36199
GbCYP40-3	GOBAR_DD26754	GbCYP43-1	GOBAR_DD26812
GbCYP41-1	GOBAR_AA27859	GbCYP39-2	GOBAR_DD34612
GbCYP41-2	GOBAR_AA25604	GbCYP66-2	GOBAR_DD21100
GbCYP41-2	GOBAR_AA25604	GbCYP49-1	GOBAR_DD27655
GbCYP42-1	GOBAR_AA16016	GbCYP39-1	GOBAR_DD36199
GbCYP42-1	GOBAR_AA09401	GbCYP40-3	GOBAR_DD26754
GbCYP42-1	GOBAR_AA09401	GbCYP43-1	GOBAR_DD26812
GbCYP48	GOBAR_AA12952	GbCYP43-3	GOBAR_DD04774
GbCYP61	GOBAR_AA36607	GbCYP40-2	GOBAR_DD36127
GbCYP61	GOBAR_AA36607	GbCYP39-1	GOBAR_DD36199
GbCYP66-2	GOBAR_DD21100	GbCYP49-1	GOBAR_DD27655
GbCYP72	GOBAR_AA31583	GbCYP62	GOBAR_DD27614
GbCYP142	GOBAR_AA09575	GbCYP26-2	GOBAR_DD13607
