# Supplementary material for: Genome-Wide Identification of Cyclophilin Gene Family in Cotton and Expression Analysis of the Fibre Development in Gossypium barbadense
Source: Int J Mol Sci. 2019 Jan 16;20(2):349. doi: 10.3390/ijms20020349 (PMC6359516; doi:10.3390/ijms20020349)
Supplement: Supplementary file 1 [file ijms-20-00349-s001.zip › ijms-423111-supplementary/Additional File 9ú║Table S5 The response cis-acting elements in GbCYPs promoters..pdf]

Additional File 9: Table S5 The cis-element analysis of GbCYPs promoters

Gene Name	light responsive element															
	3-AF1 binding site	ACE	AE-box	ATCT-motif	Box 4	chs-CMA1a	GA-motif	GATA-motif	G-box	GT1-motif	I-box	LAMP-element	MRE	Sp1	TCCC-motif	TCT-motif
GbCYP8						1573 +	293 -	54 -		455 +,418 -						271 +
GbCYP14-1					1643 -,338 +,532+, 1494 -,1236 -,1243 -	1357 -				1092 +,1828 -, 1532 +,1134 +	45 +		1089 -		50 -	1113 +
GbCYP14-2					211 +,1895 -,794 +		26 -	538 +	1913 -	1590 +,1590 +						712 -
GbCYP15				1921 -	1035 -,1062 -,185 +		1319 +	1962 -			1962 +,1960 -					738 +, 1168 +
GbCYP16-1				685 -, 680 -						1284 +	936 -				902 +, 850 +	
GbCYP16-2																
GbCYP18-1			833 +		1885 -				1200 -, 1800 -	814 +,1071 -						
GbCYP18-2	1038 -				145 +,1893 -,618 +, 276 +,726 +	410 -			1911 -							537 -
GbCYP18-3	1580 -		490 +, 779 +, 828 -	1857 -	1383 -,1972 -,1523 -, 111 +,99 +	529 -			1887 -	144 -,142 +, 124 +,168 -,23+, 167 -,175 -			852 -			
GbCYP18-4				572+, 1342+,	1296 -,4 +,761 +, 657 +,778 +		1399 +		1933 -	1905 +,326 -,35 +						235 -
GbCYP18-5	401 +		901 +	1249 +	495 +,652 +,632+, 1555 -,432 +,846 +			13 -	1886 -	1859 +,1020-, 1019 -,160 -		673 -				1070 +, 69 -
GbCYP18-6	684 -	1484-		301-,1051+	1360 -,592 +,355 +	618 -		130 -	706 +,760 -		1193 +,678 +, 665 +					108 +
GbCYP18-7			992 +	1131 +	1647 -1439 -610 +	812 +			1533 +				1279 +			294 +, 1198 -
GbCYP18-8				1916 -	1035 -			1957 -, 1328 -		1591 +	1957 +,1955 -					728 +
GbCYP18-9						198 +					943 -					
GbCYP19-1					1661 -,449 +,1308 -, 1531 -,445 +,1343 -, 1178 -,18 +,871 +	307 +928 +	1057 -,99 -	389 -		826 -,311 -,459 +			282 +			
GbCYP19-2			736 +		287 +,737 +,583 +, 525 +,637 +			1610 +, 799 -	1968 +, 1967 -	1577 -		1592 -				2288 -, 1965 +
GbCYP19-3	834 -			322 +,1380 -	1592 - ,1435 -, 1879 -,1693 -					1307 -,1306 -, 588 +						
GbCYP20-1					1865 -,1738 -,665 +	1432 -,1882-		1096 +, 361 +		1760 +,1462 +,597 +,356 +						

Gene Name	light responsive element															
	3-AF1 binding site	ACE	AE-box	ATCT-motif	Box 4	chs-CMA1a	GA-motif	GATA-motif	G-box	GT1-motif	I-box	LAMP-element	MRE	Sp1	TCCC-motif	TCT-motif
GbCYP20-2				1700 -	1714 -,610 +,1842 -	1859 -				1399+,542+,307+			539 -			
GbCYP20-3			897 +		943 +,59 +,236 +, 898 +,520 +			1040 -		780 -,781 -			754 -			
GbCYP21-1		298 -			938 +,1762 -,222 +, 380 +			2036 +, 277 -		243 -,242 -,558 +						148 -, 480 +
GbCYP21-2					676 +,1984 -,1236 -, 991 +,1968 -		1045 +			547 -,548 -						
GbCYP21-3			1207 -		1249 -,951 +,1998 -, 1188 -,1982 -					541 -						
GbCYP21-4			1818 -		1002 +,1429 -	1290 +										
GbCYP22		91 -			426 +,349 +,590 +, 409 +				5 -		1219 ,-881 -				1348 -, 1224 +	1337 +
GbCYP23				1519 +	356 +,1033 -,895 +		744 +		1228 +	591 +,153 +	1200 +					
GbCYP24-1		779 +	857 +	1693 -	1523 -,1759 -,140 +, 1781 -,984 +,23 +, 2000 -,1680 -	544 +,825 +, 333 +				1406 -,128 -, 127 -,1405 -			1472 -			1646 +
GbCYP24-2		370 +	1175 +	1641-,1804+	832 +,808 +	821 +		80+,1915-		1687 -,328 +	1410 +		25 -		1456 +	61 +
GbCYP26-1				139 +	1162 -1158 -847 +779 +1614 -1507 -1618 -			694 +	1423 -	1580 -						
GbCYP26-2	1597 -		1978 -		158 +		1242 -		1044 +,377-, 277 +,375 +	1458 -						
GbCYP26-3			897 +, 1930 -							1748 -,1749 -						
GbCYP26-4				1424 +	1065 +,45 +,1967 -, 1971 -				2532 +	2662 -,486 +, 2398 -	2496 +					979 -
GbCYP27-1			1210 +		24 +,970 +,298 +, 221 +,111 +			1967 -, 650 +	550 - ,551 +	1739 -,275 -, 405 +	1446 +,310 +, 1376 +			2036-	1492 +	
GbCYP27-2				1693 - , 1855 +	1764 -,1575 -,1821 -			1374 -, 764 -	1358 -,748 -	151 -			153 +	2023-		
GbCYP28					745 +,880 +			465 +	1005 +	661 +						609 +
GbCYP29	1554 -				144 +,200 +	1466 -,934 -, 890 +,1422+	399 +		872+,1404+, 865 +,1397 -	164 -,1782 -, 1437 +,464 -,			1784 +			
GbCYP34								1786 -	1696 -		1786 +					947 +
GbCYP36	351 -, 38 -, 378 -,				1685 -,587 +,129 +, 1467 -,442 +,1454 -, 274 +,1705 -				331 +,644 +	1574 +				1854-		1308-, 143-,456-, 1040 -

Gene Name	light responsive element															
	3-AF1 binding site	ACE	AE-box	ATCT-motif	Box 4	chs-CMA1a	GA-motif	GATA-motif	G-box	GT1-motif	I-box	LAMP-element	MRE	Sp1	TCCC-motif	TCT-motif
GbCYP37-1					125 +,1776 -				1918 -				1248 -			
GbCYP37-2		2439-					1074 +	437 -	2595 +							
GbCYP37-3	329 +				299 +,1229 -			1901 -			1901 +					1059 +
GbCYP37-4	1717 -			994 -	1926 -,1861 -,1827 -, 192 +,1237 -,1854 -, 1064 -,1650 -		1226 -			454 -,1029 +,455 -	1563 -					236 -
GbCYP37-5		1887 -		886 -	1866 -,1534 -,1751 -, 1738 -,1126 -,358 +, 53+,1674-,731+,955+	702 +	1115 -			921 +,322 -,321 -	1447 -			1926 -		97 -
GbCYP38			147 -		500 + 1779 -	1618 +			1920 -						240 -	1650 +
GbCYP39-1			1265 -, 300 +		1502 -,1373 -,837 +, 751 +,229 +,1057 -	1003 +		1553 -		1690 -,1689 -, 1491 -,1335 -	1168 +					1531 -, 1659 +
GbCYP39-2				531 +	1528 -,1113 +,557 +		174 -		979 -,123 -				729 +			
GbCYP39-3			330 -		654 +,294 +,563 +				1811 +	925 +,311 +	1003 -,1389 +				463 +	
GbCYP39-4			912 +	170 -				150 -	1072 -, 826 -	1140 +,221 +	177 -,822 +				670 -, 962 -	1814 -, 1167 +
GbCYP40-1			1319 +	474 +	1291 -,1116 -, 276 +,429 +		627 -	1586 +		1346 -,1345 -, 721 -,722 -						
GbCYP40-2			286 -	1358 +	413 +,1582 -	231 +		1418 -, 1335 -		876 -,875 -	1416 -,1418 +		469 +	690 -		1509 +
GbCYP40-3				811 -,783 +	1451 -,80 +,142 +, 112 +,198 +			1163 -	614 +	1659 -	1161 -,1163 +	551 -				
GbCYP41-1				762 +				2426 -, 762 -		346 -,908 +	52 +,174+, 3371 +,2426 -					2453 -, 356 +,
GbCYP41-2			1303 +, 1319 -	1256 -	258 +,1222 -,280 +					1247 +,1092 -, 90 +,1725 +	1415 -		87 -		2032 -	1430 +
GbCYP42-1			175 -	1390 +	1614 -	120 +		1450 -		1012 -,1013 -	1450 +,1448 -					
GbCYP42-2					1719 -,1810 -				533 -	695 +,809 +						587 -
GbCYP43-1				421+,1062+	157 +				566 -	187-,1583-, 615 -,616 -,124 +			1585 +		1829 +	
GbCYP43-2	514 -		570 -, 1082 +	1036 +				105 -	420 +	588 -,1543 – 1817 -,589 -	105 +,103 -				1811 +	1747 -
GbCYP43-3					1692 -,390 +,1816 -				1716 -,25 -	1889 -,1094 -, 1093 -			1773 -	277 -		1299 +, 951 +
GbCYP47-1	789 -		1292 +		1815 -,1609 -,1506 -		798 -	650 -	921+,1036+, 918 -	2017 +	1219 -				1878 -	1213 -

Gene Name	light responsive element															
	3-AF1 binding site	ACE	AE-box	ATCT-motif	Box 4	chs-CMA1a	GA-motif	GATA-motif	G-box	GT1-motif	I-box	LAMP-element	MRE	Sp1	TCCC-motif	TCT-motif
GbCYP47-2					1061 -,228 +,374 +, 190 +,1237 -,240 +		1049 +									
GbCYP48			1579 -		436 +,1868 -,744 -, 430 +,257 +				476 -,1768 -	441 -,440 -						46-,1346+ ,1916-
GbCYP49-1					1264 -,292 +,1472 +	456 -			1473 +	1439-,1312+, 1159-,190+			187 -	273 +	2026 -	1450 +
GbCYP49-2					1905 -,1818 -				2021+,1970+, 652-,2022 -	917 +,803 +						1216 -, 699 -
GbCYP58			1560 -		1237 -								1989 -	358 -	212 +	
GbCYP61		176 +	674 +, 633 +		1258 -,880 +,1800 -, 534 +,1485 -	1431 +		1945 -, 1594 +, 238 -	301 -	1918 -,1809-, 5 +,1589 +, 1761 -,1760 -	1596 +,1945 -	1116 -				
GbCYP62				870 -	963 +,1946 -,833 +, 289 +,608 +					1489 -,244 -, 415 +						
GbCYP63				2041 -,347 +	834 +,2004 -,824 -	1251 -			1306 -							
GbCYP66-1	1022 -	396+, 283-	767 -	1958 -	376 +,143 +							1112 +	1531 -			
GbCYP66-2			899 -		314 +,1333 -,173 +, 1161 -,364 +,535 +			1523 -								
GbCYP70			198 +	1909 -	1872 -			182 -		476 +	243 +,180 -, 182 +	543 +, 1059 +		669 +		177 +
GbCYP72				825 -	1914 -,788 +,599 +, 564 +				474 -	1459 -,867 +, 83 -						848 -
GbCYP77		676 -, 372 -, 483 +	780 +	1946 -	233 +,1909 -,721 +								1034 -, 1516 -			
GbCYP79					204 +,1324 -,1378 -, 39 +,1159 -,258 +		734 -			1824 +,1290 -, 1785+,170-,45 4+,685 +						
GbCYP142					1684 -,1598 -,701 +					994 -,726-, 725 -,993 -	535 +,910 -					

Gene Name	biotic and abiotic stress			metablism and development									hormone responsiveness					
	LTR	MBS	TC-rich repeats	ARE	CAT-box	Circadian	GCN4_ motif	HD-Zip 1	MBSI	MSA-like	O2-site	RY-element	ABRE	AuxRR-core	GARE-motif	CGTCA-motif	P-box	TCA-element
GbCYP8	1302+,552 +	34-,1354-, 747-,352-		1659 -			823 -			138+	1038 +			1591 -	157 -			595 -
GbCYP14-1		2019 -		125 -	408 -, 303 -												1967+	
GbCYP14-2		1232 +		20 -									1911-,1914+, 1913-					
GbCYP15	1915 +			13-,1491+										755 +		1124+, 1943+		
GbCYP16-1				5 -,733 +	1625 -						612 +		207 -		745 -		746 -	
GbCYP16-2																		172 +
GbCYP18-1		838 +, 137 -	402 +	1874+,988-,1289-, 1394 -,1171 +	208 -	1482+					1547 +		1801 +,1201 +, 1800 -		121 -			786 -, 1119 +
GbCYP18-2				957 +,399 -									1912 +,1911 -					
GbCYP18-3	221-,138-, 120-,179 +			1050 -,1961 +									462 -,754 +, 1888 +,1887 -	435 -		1572 +	663 -	394 -
GbCYP18-4				559 -,1174 -	1008 -								1934+,1933-, 1931-					
GbCYP18-5			1066+, 749-	292 -,1095 -,454 -	930 -								1887+,1886-, 1884+					
GbCYP18-6	643 -		1509+	183 -,1912 +									706 +,761 +, 707 +	800 -				794 -
GbCYP18-7	165 -		290 +	1963 +			56 -				821 +		1533 -	886 -				209 -
GbCYP18-8	1910 +			290 -,1487 +,880 +				1050-			1935 -			1083 -				
GbCYP18-9				738 +	1650-, 873+												1115+	
GbCYP19-1				507-,1645-,1639+, 1166 -												975+, 1276-		1715 -
GbCYP19-2		2021+, 1249 -	1261 +	656 +,242 -									1969 +	2167 +				
GbCYP19-3	2060 -			352 -		703 -							1174 -,770 -		176 -		986 +	
GbCYP20-1				1239 +,1785 +	1312-, 1221+											704 +	1093 -	284 +
GbCYP20-2		1270 +		1176 +	1158 +										1010+		1030 -	
GbCYP20-3	931 -															450-, 2020 -		
Gene Name	biotic and abiotic stress			metablism and development									hormone responsiveness					

	LTR	MBS	TC-rich repeats	ARE	CAT-box	Circadian	GCN4_motif	HD-Zip 1	MBSI	MSA-like	O2-site	RY-element	ABRE	AuxRR-core	GARE-motif	CGTCA-motif	P-box	TCA-element
GbCYP21-1	1003 -,1957 +,1044 +			809 -,1393 +,253 +, 26 +								1416 -			742 -		1302+	1622 +
GbCYP21-2		1393 +		101 +,1737 -												176 +, 1353 -		
GbCYP21-3	191 +	1406 +		1750 -,485 -,194 +														414 +
GbCYP21-4				1085 -,1185 +										1463 -	132 -	1983 -		942 -
GbCYP22			1333 +							1385+			6 +,294 +	1080+				
GbCYP23	68 +	1915 +		2007 +	384 -								1228 -					
GbCYP24-1		1707 -			15 +									76 +		823 +, 746 +	1911-, 372+	1852 -
GbCYP24-2		1590 -		1809 +,1138 +, 209 -,1955 +, 1265 +,1554 +												1198 + 281 +		
GbCYP26-1				1363 +,363 +,40 -							1420 +		1424 +					
GbCYP26-2	217 +	1551+		1915 -									1105 +,378 +, 1044 -,338 +, 318 -,1064 +	1216 -, 153 +				
GbCYP26-3	208 -,524 -			1515 -														
GbCYP26-4		628 +	979 -	1655 +,1328 -,523 -	2002 +						856 -, 2240 +, 919 +		2532 -,2244 +		740 +	2242 -, 2230 -	835 +	2715 +, 1181 -
GbCYP27-1	1162 +		1987 +	2017 +,1300 +, 1860 +,2007 +									552 +		308 -			
GbCYP27-2			164 -	1936 +,631 +, 1241 +				1410-			310 -, 920 -		1953+,749+,478- ,1088 -,1359 +					
GbCYP28	303 -			1163 +							1670 +, 364 +		1003 -,1005 -		597 +	1441 -		
GbCYP29											1807 +		1404 -,1398 +, 1397 -,872 -, 865 +,1822 -, 866 +	1857 +		579 +, 1111+, 1824 +		452-, 1756-, 984-, 1963 +
GbCYP34				1298 +,259 +,55 +,1260 -,867 +							43 -		1697 +,72 -					1666 -
GbCYP36	1730 +			712 -,1475 -		931 +	1269+	1503-			764 +, 908 +		332 +,645 +					
GbCYP37-1		810 +	903 -	1089 -,361 +,100 -	228 -, 1133 -							1817 -	284 +,1919 +				1555+	
Gene Name	**biotic and abiotic stress**			**metablism and development**									**hormone responsiveness**					

	LTR	MBS	TC-rich repeats	ARE	CAT-box	Circadian	GCN4_motif	HD-Zip 1	MBSI	MSA-like	O2-site	RY-element	ABRE	AuxRR-core	GARE-motif	CGTCA-motif	P-box	TCA-element
GbCYP37-2				1505 -									2596 +					
GbCYP37-3			1280+	177 +,265 +, 1409 +,986 +											1578 -		1715+	1782 -
GbCYP37-4	96 +,85 +	957+,290- ,1132 +		1167 -										946 -, 184 +				
GbCYP37-5		151 -		1056 -		1435+			1021+		1843 +			838 -,45 +				
GbCYP38				726 +,475 -	1450-, 188-								1921 +					
GbCYP39-1		407 +		809-,1947-		1604 -					171 +			1101 +			1816+	
GbCYP39-2		83 -		213+,278+,1032 -			581 +				1680 -		980 +				2064 -	852 +, 1270 +
GbCYP39-3		1385 +		1554 +,416 -, 116 -,1992 +							1245 -		1811 -					1567 -
GbCYP39-4	1808 +, 1042 +			1633 +,604 -	1002 -						1132 +, 766 +		827 +	1609 +	384 +			
GbCYP40-1		97 -		1234+,808+													1595 -	880 -
GbCYP40-2	1007 +			1953-,1050+,273+	653 +		1165+							1907 +	208 -		1349 -	
GbCYP40-3				1608 +,85 -	56 -	1719+					1114 +		614 +,615 +,43 -					266 +
GbCYP41-1					2846 -, 2972 -, 3197 -									391 -		2720-, 2837-		319+, 3516+, 2815 +
GbCYP41-2	1096+		1146-			547 +								814 -		888 -, 817 +		1009 +
GbCYP42-1	1347 +	97 +		1985 -,753 +,874 -			1949+, 1198+										381-, 1505+	1570 +
GbCYP42-2	1682 -,752 +	943 +		446 +,631 +,1595 -, 488 -,755 +							1429+, 1283-	222 +	1926-,534+, 1875-	1984 +		913 -		876 -, 1561 -
GbCYP43-1	1327 -,1989-			762 +,958 +,1864 +		993 +					1128-, 1505+		567 +		1768 -		1973 -	
GbCYP43-2	1970 -	1660+, 1800+		735 +,122 -,931 +, 1846 +		966 +					1130-, 1465+		1171 -,420 -				1954 -	
GbCYP43-3	1854 +		1309 +			421 -							1717 +	989 +				1559 -
GbCYP47-1	939 -,584 +		1213-, 481+	1013 -,37 -	1729 -			312 +					922 +,1036 -, 600 -,921 +					1060 +
GbCYP47-2		852 +		416 -,1199+,807-							504 +							1525 +
GbCYP48	1903 +		10+, 1356+										1769 +,477 +					394 +

Gene Name	**biotic and abiotic stress**			**metablism and development**									**hormone responsiveness**					
	LTR	MBS	TC-rich repeats	ARE	CAT-box	Circadian	GCN4_ motif	HD-Zip 1	MBSI	MSA-like	O2-site	RY-element	ABRE	AuxRR-core	GARE-motif	CGTCA-motif	P-box	TCA-element
GbCYP49-1	1842+		1213 -	1368 +,366 -	10 -	613 +							1473 -		694 +			1074 +
GbCYP49-2	1781 -	1051 +		1704-,863+,607-, 565 +,2000 +	520 -						1391-, 1534+	341 +	2022-,1970-, 653+	2080 +		1021 -		25 +
GbCYP58				955 -,521 -,69 +											1565+			1645-, 215+, 234+
GbCYP61				1230 -		1996 -							1736 -,302 +	971 +		971 -		
GbCYP62		1398+		947 -,941 +,502 -	1686 -								359 -					
GbCYP63			1643 +				1214 -						1307 +					1607+, 650-
GbCYP66-1	989 +		1575 +	1276-,1013-, 1324-					410 -							1802 -		1538 +
GbCYP66-2				150 -,562 -							649 +							981 -
GbCYP70	942 +		1511 +	455+,1270-,775+	697 +									14 -		1816 -		1475 +
GbCYP72	1174 +	1354+	54 +	893 +,422 -,899 -	1656 -				1559+				474 -,197 -	228 -	151 +	231 +		963 -
GbCYP77	974 +		1560 +	126-,1309-,1291-				1502-										1523 +
GbCYP79				1009 -		900 +												
GbCYP142	463 +,980 +			870 +,1012 +										1038 +			1483 -	

3-AF1 binding site(CACTATCTAAC), ACE(CTAACGTATT), AE-box(AGAAACAA), chs-CMA1a(TTACTTAA), GA-motif(ATAGATAA), GATA-motif(GATAGGG), G-box(CACGTC), GT1-motif(GGTTAA), I-box(AGATAAGG), LAMP-element(CTTTATCA), Sp1(GGGCGG), TCCC-motif(TCTCCCT), TCT-motif(TCTTAC), ATCT-motif(AATCTAATCC), Box 4(ATTAAT), MRE(AACCTAA), LTR(CCGAAA), MBS(CAACTG), TC-rich repeats (ATTTTCTTCA), ARE(AAACCA), CAT-box(GCCACT), circadian(CAAAGATATC), GCN4_motif(TGAGTCA), HD-Zip 1(CAAT(A/T)ATTG), MBSI(TTTTTACGGTTA), MSA-like(TCCAACGGT), O2-site(GATGA(C/T), RY-element(CATGCATG), ABRE(CACGTG), AuxRR-core(GGTCCAT), GARE-motif(TCTGTTG), P-box(CCTTTTG), TCA-element(GAGAAGAATA), CGTCA-motif(CGTCA).
