# Supplementary figures and images for: Genome-Wide Identification of Cyclophilin Gene Family in Cotton and Expression Analysis of the Fibre Development in Gossypium barbadense
Source: Int J Mol Sci. 2019 Jan 16;20(2):349. doi: 10.3390/ijms20020349 (PMC6359516; doi:10.3390/ijms20020349)

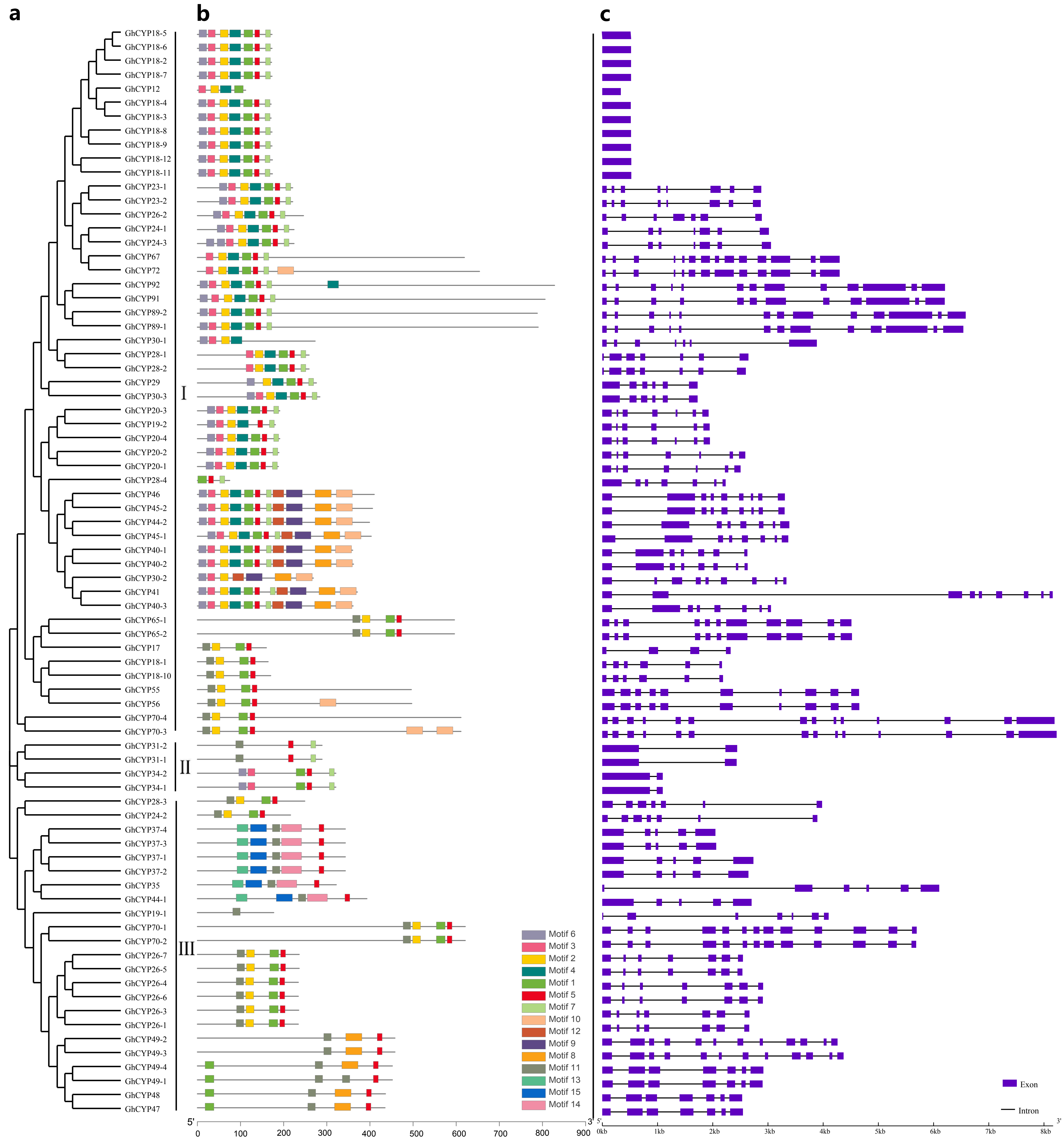

Supplement: Supplementary file 1 [file ijms-20-00349-s001.zip › ijms-423111-supplementary/Additional File 2ú║Fig. S1 Phylogenetic relationship, motif analysis and Exon-intron gene structure of the CYPs gene family in TM-1..pdf]

**a**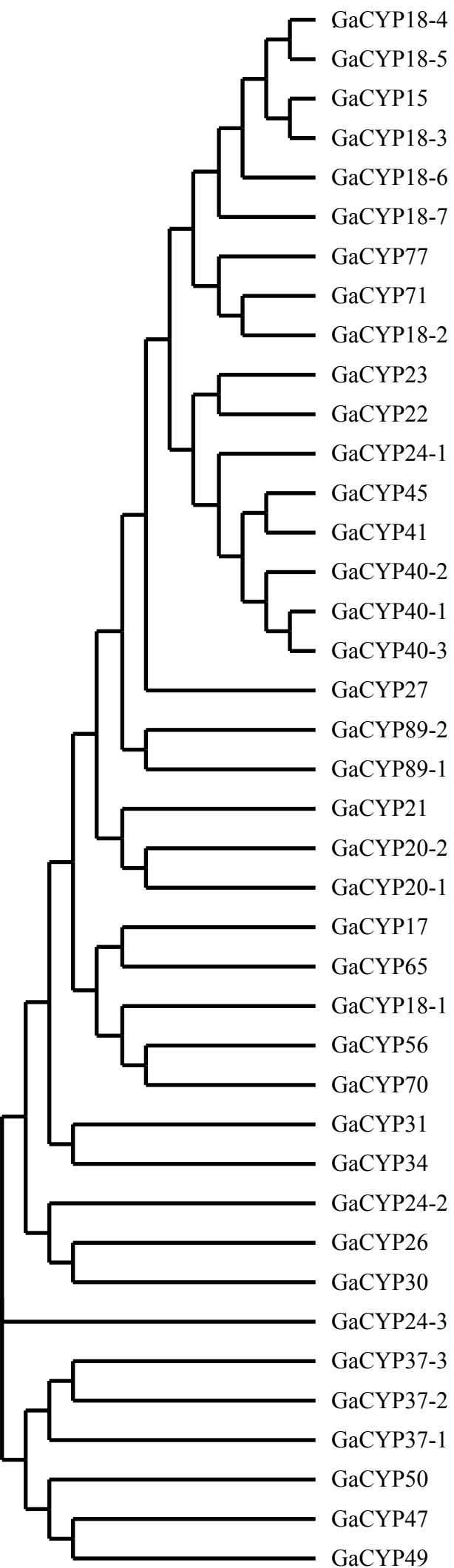**b**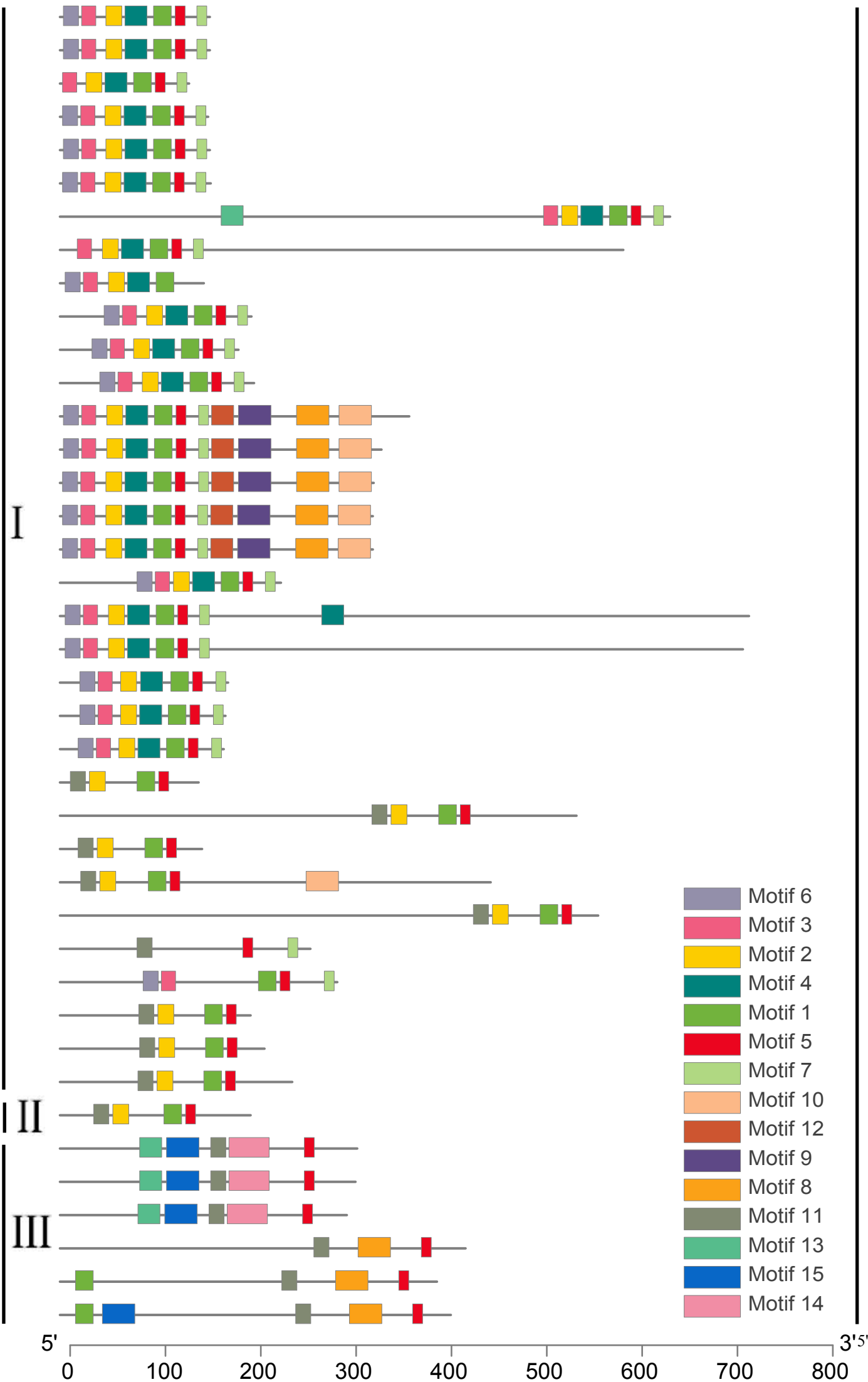**c**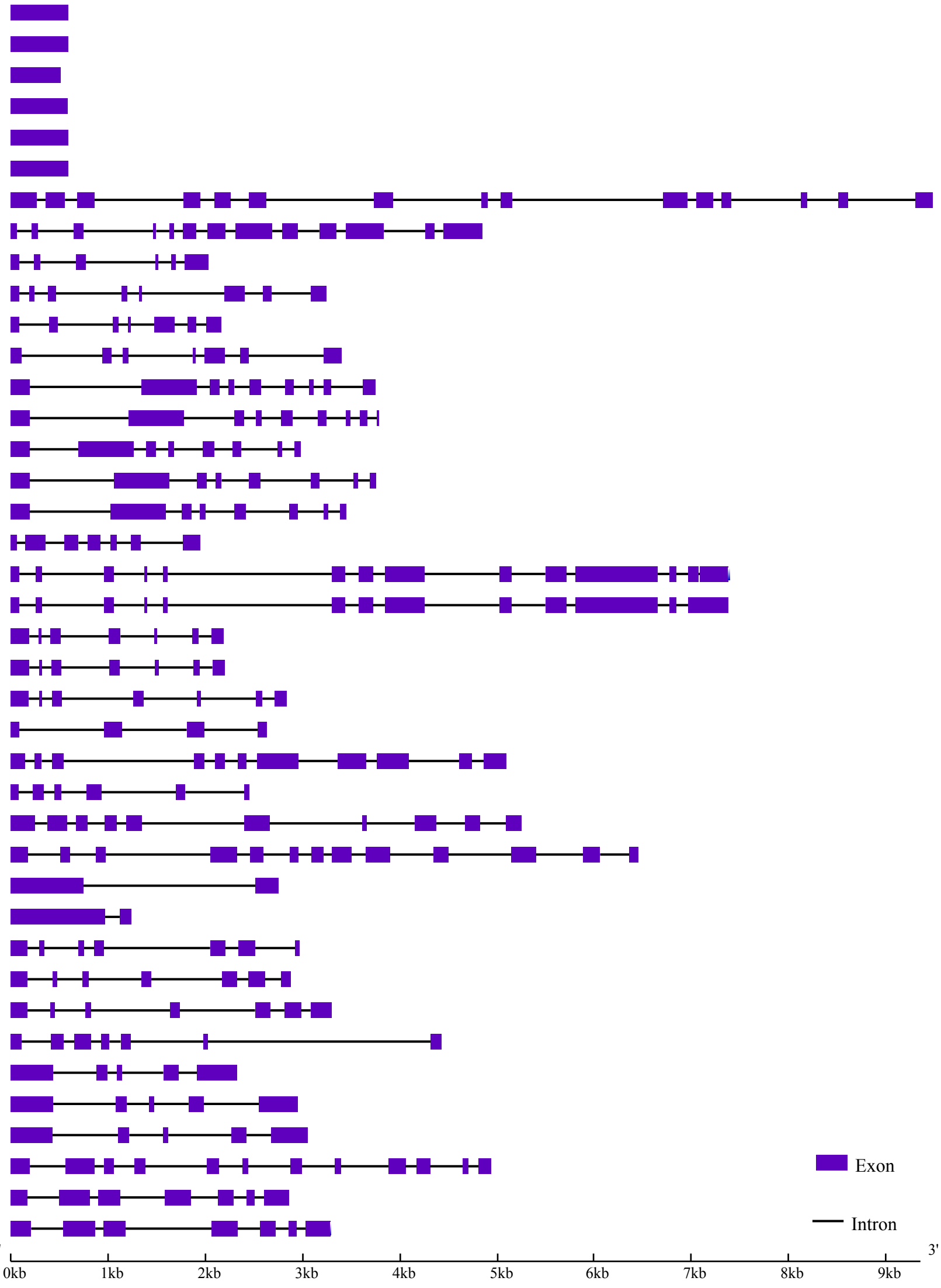

Supplement: Supplementary file 1 [file ijms-20-00349-s001.zip › ijms-423111-supplementary/Additional File 3ú║Fig. S2 Phylogenetic relationship, motif analysis and Exon-intron gene structure of the CYPs gene family in G. arboreum..pdf]

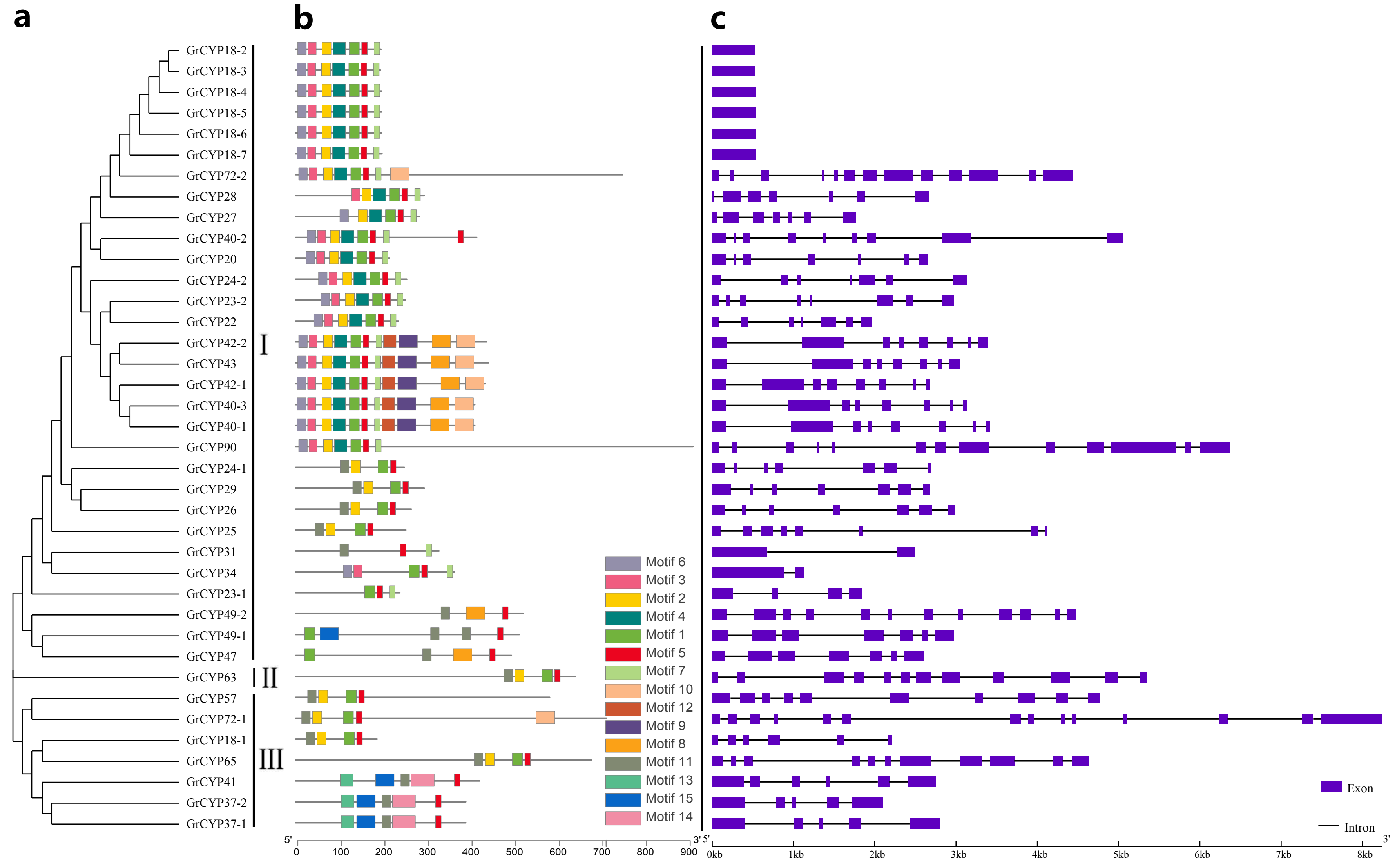

Supplement: Supplementary file 1 [file ijms-20-00349-s001.zip › ijms-423111-supplementary/Additional File 4ú║Fig. S3 Phylogenetic relationship, motif analysis and Exon-intron gene structure of the CYPs gene family in G. raimondii..pdf]

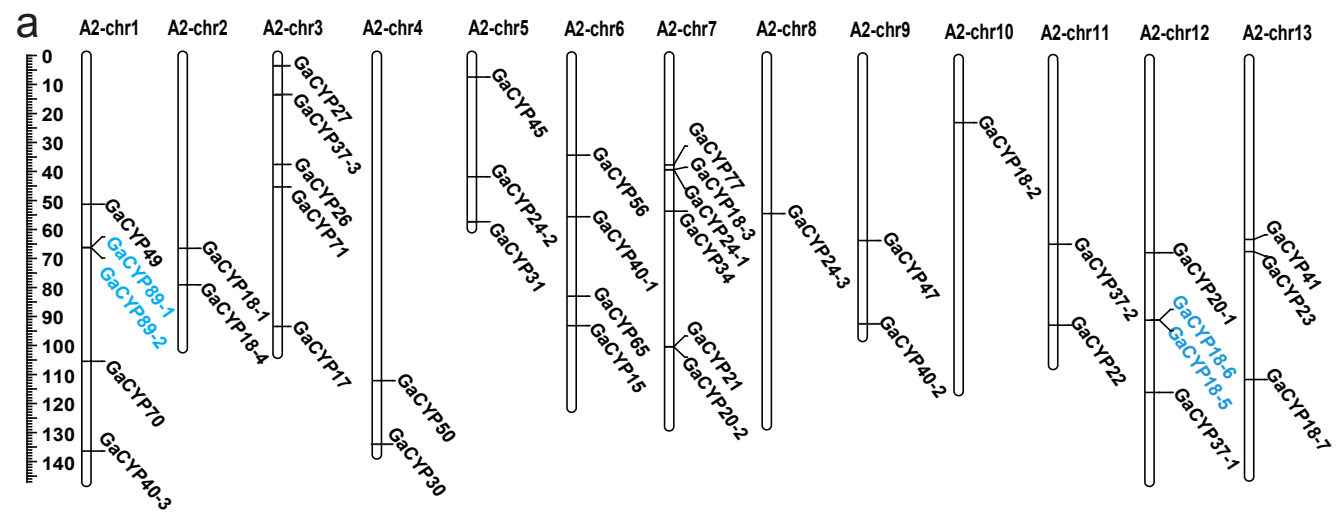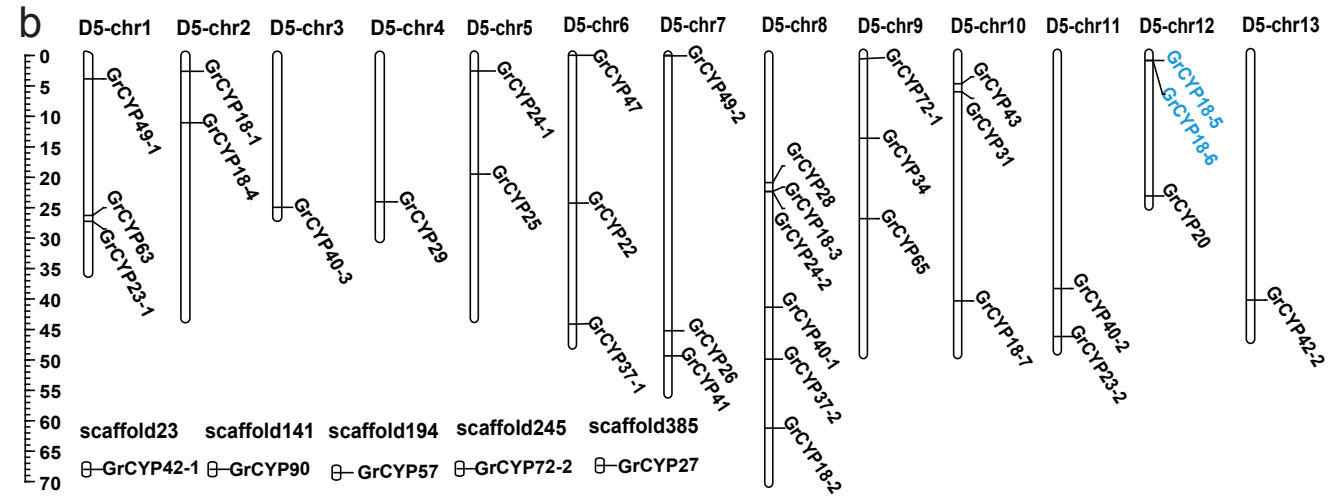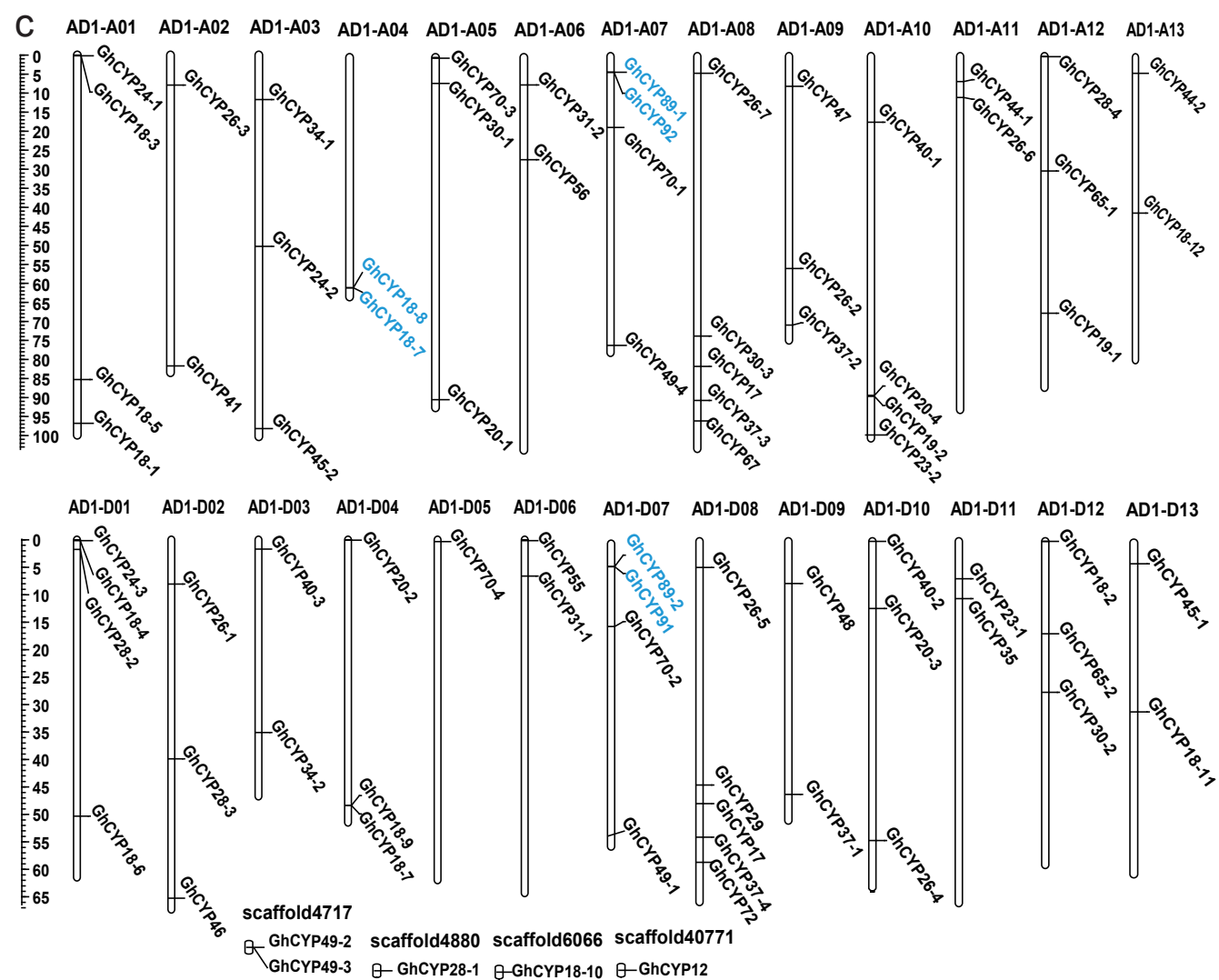

Supplement: Supplementary file 1 [file ijms-20-00349-s001.zip › ijms-423111-supplementary/Additional File 6ú║Fig. S4 Chromosomal localization of GhCYP,GaCYP,GrCYP genes..pdf]
